# Supplementary material for: Early Depletion of Neutrophils Reduces Retinal Inflammation and Neovascularization in Mice with Oxygen-Induced Retinopathy
Source: Int J Mol Sci. 2023 Oct 27;24(21):15680. doi: 10.3390/ijms242115680 (PMC10648252; doi:10.3390/ijms242115680)
Supplement: Supplementary file 1 [file ijms-24-15680-s001.zip › ijms-2627643-supplementary.pdf]

Early Depletion of Neutrophils Reduces Retinal Inflammation and Neovascularization in Mice with Oxygen-Induced Retinopathy

Devy Deliyanti, Varaporn Suphapimol, Phoebe Ang, Xiuying Tang, Abhirup Jayasimhan and Jennifer L. Wilkinson-Berka *

Department of Anatomy and Physiology, School of Biomedical Sciences, University of Melbourne, Parkville, VIC 3010, Australia

* Correspondence: jennifer.wilkinsonberka@unimelb.edu.au

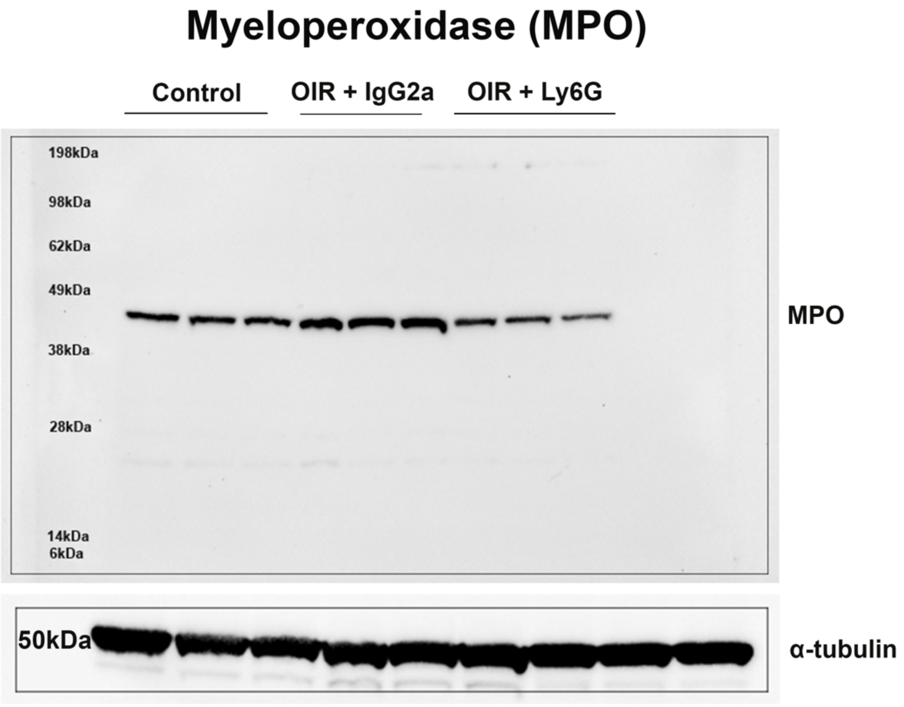

Figure S1. Uncut western blot image of myeloperoxidase (MPO), showing the distinct bands that correspond to MPO protein expression. The lower panel shows the α-tubulin loading controls.

Table S1. Body weight of room air control and OIR mice at various time points.

Group	Postnatal Day (P)	*n*	Body Weight (g)
Room air control	P9	7	6.48 ± 0.69
OIR control	P9	7	5.08 ± 0.39
Room air control	P18	7	7.85 ± 0.70
OIR control	P18	7	6.35 ± 0.65 **
Room air control	P12	18	6.60 ± 1.03
OIR + IgG2a mAb	P12	18	5.88 + 0.24 *
OIR + anti-Ly6G mAb	P12	18	6.03 +0.87 *
Room air control	P13	12	6.21 ± 0.94
OIR + IgG2a mAb	P13	12	5.39 ± 0.77 *
OIR + anti-Ly6G mAb	P13	12	5.22 ± 0.62 **
Room air control	P18	18	8.2 ± 0.62
OIR + IgG2a mAb	P18	18	6.92 ± 1.68 ***
OIR + anti-Ly6G mAb	P18	18	6.53 ± 0.50 ***

* $p < 0.05$, ** $p < 0.01$, *** $p < 0.001$ to room air control (one-way ANOVA followed by Tukey's multiple comparisons test). All values are mean ± SEM.
